# Operando Measurement of Transition Metal Deposition in a NMC Li‐Ion Battery Using Laboratory Confocal Micro‐X‐ray Fluorescence Spectroscopy

**DOI:** 10.1002/smll.202502460

**Published:** 2025-04-18

**Authors:** Ioanna Mantouvalou, Lena Mathies, Katja Frenzel, Yannick Wagener, Leona Johanna Bauer, Daniel Grötzsch, Matthias Müller, Birgit Kanngießer, Martin Winter, Sascha Nowak, Adrian Jonas, Burkhard Beckhoff

**Affiliations:** ^1^ Helmholtz‐Zentrum Berlin für Materialien und Energie Albert‐Einstein‐Str. 15 12489 Berlin Germany; ^2^ Physikalisch‐Technische Bundesanstalt (PTB) Abbestr. 2–12 10587 Berlin Germany; ^3^ Technische Universität Berlin Germany Hardenbergstr. 36 10623 Berlin Germany; ^4^ MEET–Münster Electrochemical Energy Technology Corrensstraße 46 48149 Münster Germany

**Keywords:** confocal micro X‐ray fluorescence, CR2032 coin cell, NMC Li ion batteries, non‐destructive testing, operando elemental investigations

## Abstract

The degradation of batteries has very different causes depending on the material and operation modes. However, most of these causes are associated with changes in one or more interfaces, in particular through depositions and their potential chemical changes under operating conditions. Over the last decade operando investigations have therefore become increasingly state‐of‐the‐art, elemental analysis of full cell systems, though, is still missing due to a lack of depth resolved methods. Using laboratory confocal micro‐X‐ray fluorescence spectroscopy the analysis of a Li‐ion battery coin cell during 10600 cycles are presented. It is shown that the confocal setup enables to differentiate between the nickel‐manganese‐cobalt‐oxide (NMC) cathode with high levels of transition metal concentration and a possible deposition of traces of Mn, Ni, Co in the underlying layers. This allows for spatially resolved insights in operando without changing the layer stack, nor electrode area. This paper is the first to demonstrate the non‐destructive and quantitative elemental analysis of battery interfaces under operating conditions. This quantitative analysis is the prerequisite for the determination of absolute transport and conversion rates, without which the transition from empirical research to a focused development of batteries will not succeed.

## Graphical abstract

Transition metal dissolution in Li‐ion batteries can lead to reduced battery performance. To monitor elemental migration during cyclization, laboratory confocal micro‐X‐ray fluorescence spectroscopy is used. The investigation of a nickel‐manganese‐cobalt‐oxide (NMC) coin cell during thousands of charging cycles shows increasing Mn deposition in the graphite anode during the first 6 weeks of cyclization.

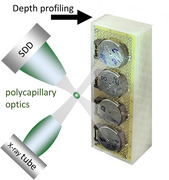

## 1 Introduction

Lithium‐ion batteries (LIB) are widely used to store electricity for portable devices. Research activities towards high performance, better stability, and safety have increased significantly over the last decades. Improving state‐of‐the‐art LIB is a highly multidisciplinary field necessitating the combination of ex situ, in situ, and operando approaches to be able to understand the complex conversion and transport mechanisms and degradation processes inside LIB.^[^ ^1^ ^]^

The cathode material has a big influence on battery performance, costs, and stability.^[^ ^2^ ^]^ Due to the high energy density, good rate capability, and reliable cycling performance of the cells,^[^ ^3^, ^4^ ^]^ the combination of layered nickel‐manganese‐cobalt‐oxides (NMC) with a graphite electrode (anode) is one of the most promising battery systems and already prevalent in electric vehicles and consumer electronics. The theoretic specific capacity is 275 mA h g^−1^, but the structural instability at high Li^+^ removal state is still one main drawback of these materials leading to reduced practical capacity.^[^ ^5^ ^]^

During the last decades the composition of NMC was constantly improved in view of higher practical capacity, better performance, and lifetime of the cells. Especially Ni‐rich NMC materials (LiNi_x_Mn_y_Co_1−x−y_O_2_, x ≥ 0.5) have been investigated intensively, showing significantly increased energy density. For the Ni‐rich NMCs specific degradation processes become more severe due to the reduced structural stability. Degradation mechanisms for NMC batteries are thoroughly reviewed in,^[^ ^4^ ^]^ and one of the main reasons for capacity fade and the decay of the cell performance is the surface degradation of active NMC particles enforcing further degradation through reactions with the electrolyte solution and leading to transition metal dissolution,^[^ ^2^, ^5^, ^6^, ^7^, ^8^, ^9^ ^]^ most strongly for manganese.^[^ ^10^, ^11^, ^12^ ^]^ The diluted transition metal (TM) can penetrate the solid electrolyte interphase (SEI) formed on the anode surface. By further reactions with the electrolyte solution active Li is consumed, the SEI thickens, and the formed (by)products change the SEI composition and its properties. Furthermore, TM species can intercalate in the graphite electrode, where the changed distance between the carbon layers block Li diffusion pathways and intercalation spots. All these processes lead to a capacity fade of the cell and reduced performance.^[^ ^13^, ^14^, ^15^ ^]^

To improve battery performance the processes happening on the surface of the active particles and/or at the interphase surface must be understood requiring sensitive investigation methods, local discrimination and long‐term monitoring.

While post‐mortem and ex‐situ studies can give valuable information, they are not fully reliable, due to the possible damage of fragile components or reaction products during the disassembly of the cells, the washing of the electrodes, and the extraction of the individual cell components. In depth investigation of the occurring processes during the cycling of the batteries is better addressed by in‐situ and operando approaches. But several challenges typically impede the conduction of the experiments. Depending on the measurement method specific entrance windows are needed, the cell set up, and measurement chambers must be adapted. Every geometrical or compositional change of the original system can lead to variation in the cell performance and chemistry and must be considered when interpreting the results.

**Figure 1:**
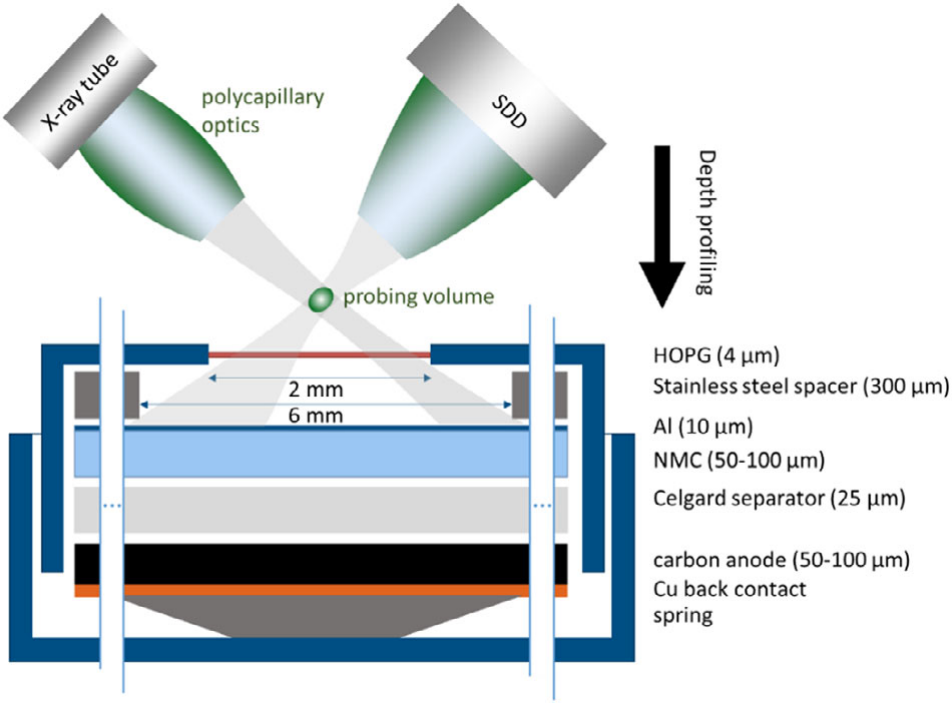
Schematic view of the cell architecture and the confocal setup with probing volume. Depth profiling measurements are conducted by measuring CMXRF spectra at positions relative to the surface.

To date, several X‐ray techniques are used to study battery materials.^[^ ^16^, ^17^ ^]^ The penetrating nature of X‐rays facilitates the investigation of full functioning cells with little to no radiation damage. Here, X‐ray fluorescence (XRF) and absorption spectroscopy (XAS)^[^ ^18^ ^]^ enable elemental and chemical specificity. This specificity is combined with 3D resolution by employing a confocal setup.^[^ ^19^, ^20^ ^]^ Through the use of a focused X‐ray beam and a polycapillary lens in front of the energy‐dispersive detector, a probing volume is formed, from which information is exclusively derived, see **Figure** **1**. By moving this volume through a sample, line scans, 2D maps and 3D volumes can be derived^[^ ^21^ ^]^ rendering 3D elemental imaging possible. Specifically, depth profiling can be achieved when moving the probing volume stepwise deeper into a sample. When absorption is low, the fluorescence line intensity profiles as a function of depth can be interpreted qualitatively. For quantification, reconstruction procedures are necessary^[^ ^22^, ^23^, ^24^, ^25^ ^]^ to extract compositions and sample geometry. When using polycapillary optics, the probing volume size decreases with increasing energy with values typically ranging between 20 µm for fluorescence lines with high energies and 50 µm for low energies, see also **Table** **1**. This depth resolution does not degrade into the depth, whereas the measured count rate decreases exponentially due to self‐absorption.

**Table 1:** Comparison of the FWHM and the maximal intensity value of depth profiles on thin foils for the two laboratory setups and the proof‐of‐principle experiment at PTB

		MiFo@PTB [low alpha]	BLiX Spectrometer	M4 Spectrometer
FWHM [µm]	Ti Kα	18	42	51
	Fe Kα	14	30	40
	Cu Kα	13	24	31
Max. intensity [cps]	Ti Kα	2	18	9.5
	Fe Kα	1.4	46	18
	Cu Kα	3.9	6	23

A confocal setup is both feasible in the laboratory^[^ ^26^, ^27^ ^]^ and with synchrotron radiation,^[^ ^20^, ^22^ ^]^ the latter offering the possibility to tune the excitation energy around an absorption edge and retrieve depth resolved XAS information.^[^ ^28^ ^]^ Confocal micro X‐ray absorption near edge spectroscopy (CMXANES) on ex‐situ cathode materials was first presented by Menzel et al.,^[^ ^29^ ^]^ demonstrating the high potential for depth resolved analysis.

The aim of this work is the operando determination of TM deposition with a focus on Mn at the SEI and/or intercalation into the carbon anode during the cyclization of a NMC battery. To enable photon‐in/photon‐out experiments, the standard stainless steel CR2032 coin cells are equipped with a HOPG (highly oriented pyrolytic graphite) window and a spacer for better pressure compensation. Notably, no modification of the cell stack design is necessary as shown in the schematic in Figure 1.

Using laboratory confocal micro‐XRF (CMXRF) we present for the first time the analysis of an intact NMC coin cell during 10600 cycles. We show that the confocal setup enables to differentiate between the NMC cathode with high levels of transition metal concentration and a possible deposition of traces of Mn, Ni, Co in the underlying layers (separator, carbon anode), see Figure 1. This is a unique feature of the confocal geometry due to the possibility to only probe atoms within the formed probing volume without contributions from other parts of the excitation and detection pathways. In conventional XRF analysis, the contributions of, e.g., Mn K fluorescence from the cathode and the underlying layers would be integrated, preventing the localization of a possible deposition.

The operando measurements using laboratory CMXRF instrumentation at the Berlin Laboratory for innovative X‐ray technologies (BLiX) render the investigation of changes in the elemental depth profiles feasible. Both post‐mortem analysis and imaging after disassembly are shown and transport mechanisms discussed. Additionally, a first proof‐of‐principle investigation at the microfocus (MiFO) beamline in the PTB laboratory at the synchrotron radiation source BESSY II is presented which demonstrates the feasibility to perform operando XANES investigation in a confocal setup.

## 2 Results

**Figure 2:**
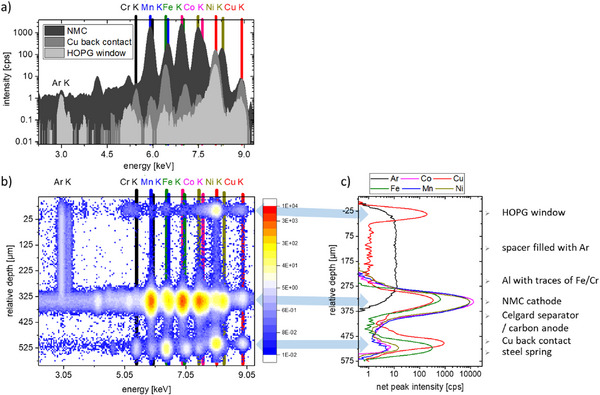
Example of an operando depth profile measurement obtained with the BLiX CMXRF spectrometer. a) CMXRF spectra at three selected depth positions: at the HOPG window, in the NMC cathode and in the Cu back contact. b) 2D map of the CMXRF spectra in the energetic region between the Ar K and Cu K fluorescence lines as a function of relative depth (intensity is given in cps). c) Depth profiles of the individual fluorescence peaks are the net peak intensities as a function of relative depth. The different layers of the NMC coin cell are attributed to the elemental signals.

**Figure** **2** shows the CMXRF depth profiling measurement of the cell in the third charging cycle at roughly the middle lateral position of the HOPG window. Exemplary XRF spectra (Figure 2a) are shown with the probing volume at depth position in the HOPG window, the NMC cathode and the Cu back contact. 2D maps of all spectra of a depth profile as a function of relative depth position illustrate the information density obtained (Figure 2b), therefore in the following the derived net peak intensities (Figure 2c) are shown as a function of relative depth position. Net peak intensities are defined as the resulting count rate after the deconvolution process (stripping of the background and fitting of the fluorescence peaks with Gaussian distributions). The fluorescence peaks can be attributed to the different layers of the cell. The Cu K fluorescence peak in the spectra is a superposition of the Cu content and the density of the material because of the elastic and inelastic scattering of the characteristic lines from the Cu X‐ray tube. The Cu intensity increases for example in the HOPG window due to scattering, but in the Cu foil as a result of scattering and fluorescence. Due to the assembly in Ar atmosphere, a gas bubble is present between the HOPG window and the Al/NMC layer. The NMC cathode and the Cu back contact are clearly distinguishable from each other, though there is no simple possibility to differentiate between carbon anode and separator. Self‐absorption is low, so that the non‐destructive elemental investigation of the full layered structure (> 500 µm) down to the steel spring through the intact NMC cathode is possible. Thus, repeated measurements of the same cell at different cycle numbers and/or states of charge (SOC) can be performed.

**Figure 3:**
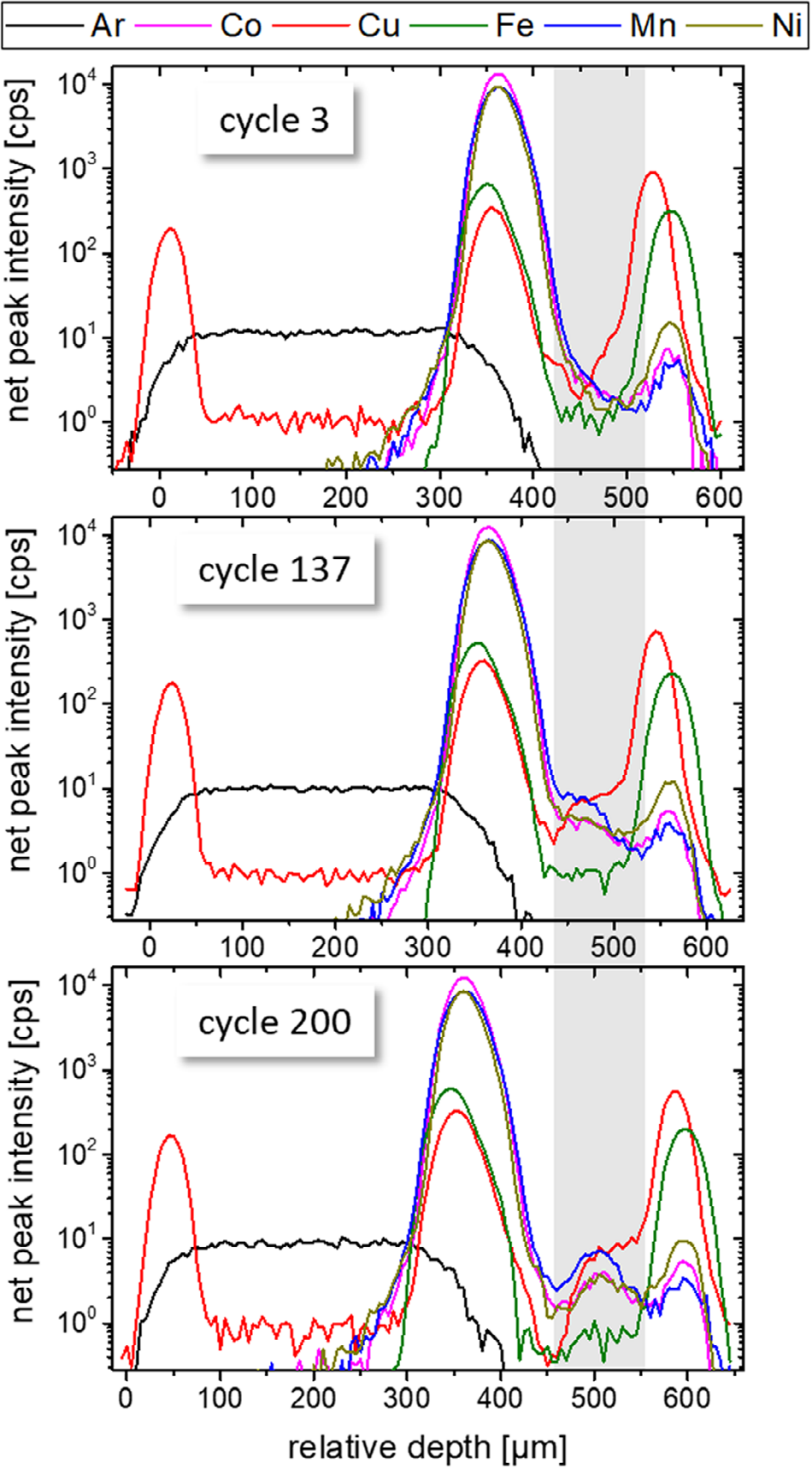
Three depth profiling measurements in the first 3 weeks (after 3d 19 h (cycle 3), 17d 21 h (cycle 136) and 22d 15 h (cycle 199)) show a clear change of shape of the TM depth profiles between the NMC cathode and the Cu back contact. The area of the separator/carbon anode is marked with grey.

After the formation cycle (at a rate of C/20 with constant current charging and a 30 min constant voltage hold at 4.2 V, followed by constant current discharge) the cell was cycled at C/10 three times followed by continuous cycling for more than 100 days at a rate of C/2. During this period the cycling settings were changed repeatedly, see **Table** **2** for cyclization parameters. The voltage minimum and maximum were changed three times to induce enhanced stress on the cell and reinforce degradation. Different constant voltage holds were introduced to assess the dependency of elemental deposition on the state of charge. In the first six weeks, sixty depth profiling CMXRF measurements with a duration of about 1 h were performed, some of them while holding the cell in a loaded or empty SOC. While the first few hundred cycles lasted about an hour to half an hour, toward the end of life of the cell, the cycle duration was reduced to a few seconds. Three example depth profiling measurements from the first three weeks are shown in **Figure** **3** where the area of the separator/carbon anode is marked with light grey. The measurements were taken in cycles 3, 137 and 200, run with rates of C/10, C/2 and C/2, and showed specific capacity values of 183 mAh g^−1^, 106 mAh g^−1^ and 72 mAh g^−1^, respectively. Changes become apparent in the TM depth profiles.

**Table 2:** Cycling parameters: After the formation cycle (at a rate of C/20 with constant current charging and a 30 min constant voltage hold at 4.2 V, followed by constant current discharge) the cell was cycled at C/10 three times followed by continuous cycling for more than 100 days at a rate of C/2.

Cycle Number	Voltage min.‐ max. Value [V]	Duration	Cycle Number	Interruptions
0–82	3–4.7	2 weeks	9	wait @ 4.7 V
83–133	3–4.2	4 days	22	hold @ 3 V
134–509	3–4.7	18 days	23	hold @ 4.7 V
510–10793	2 – 4.7	4 weeks	39	hold @ 3 V
			54	hold @ 4.7 V
			82/95/109	hold @ 4.2 V
			83/96/110	hold @ 3 V
			793	wait @3.76 V … 3.54 V

When comparing all 60 depth profiles collected in more than 100 days, it becomes apparent, that there is a slight movement of layers (up to ≈35 µm). When comparing this movement to the state of charge no significant correlation can be found. The seemingly random movement results in the necessity to always measure a full depth profile, to compare fluorescence intensities at a specific position of the stack in a layer.

**Figure 4:**
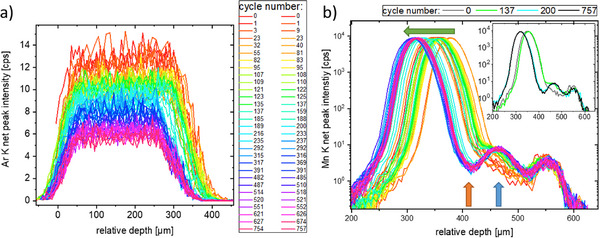
All Ar K and Mn K depth profiles over a duration of 6 weeks: a) The Ar K fluorescence depth profiles show an overall decrease of Ar and a reduction of the thickness of the Ar bubble; b). In the Mn K depth profiles a separation of the layers is visible (green arrow) with a gap forming between separator and carbon anode (orange arrow). The Mn K intensity in the carbon anode increases by a factor of 4 (blue arrow). The inset shows the first and last depth profile as well as two examples shown in Figure 3.

To further interpret the changes in intensity all depth profiles of one fluorescence peak were plotted in one graph relatively shifted to the position of the steel spring (at ≈575 µm relative depth). The results for the Ar K and Mn K fluorescence are presented in **Figure** **4**. The overall decrease of Ar and a reduction of the thickness of the Ar bubble may result from a not completely airtight cell housing. In the Mn signal (as in the Co and Ni) a gap formation between separator and carbon anode is observable, leading to an increase of distance between NMC cathode and carbon anode. Additionally, an increase of Mn (and Co and Ni) intensity in the carbon anode by a factor of 4 is detected. This increase is seen after ≈140 cycles and the final separation of the layers after ≈200 cycles. After this time, the depth profiles do not change significantly, hinting to some kind of saturation, see the inset in Figure 4, right, where additionally the last measurement is plotted. Due to this fact and the rapidly decreasing duration of the cycles, no more operando measurements were performed after about 6 weeks.

**Figure 5:**
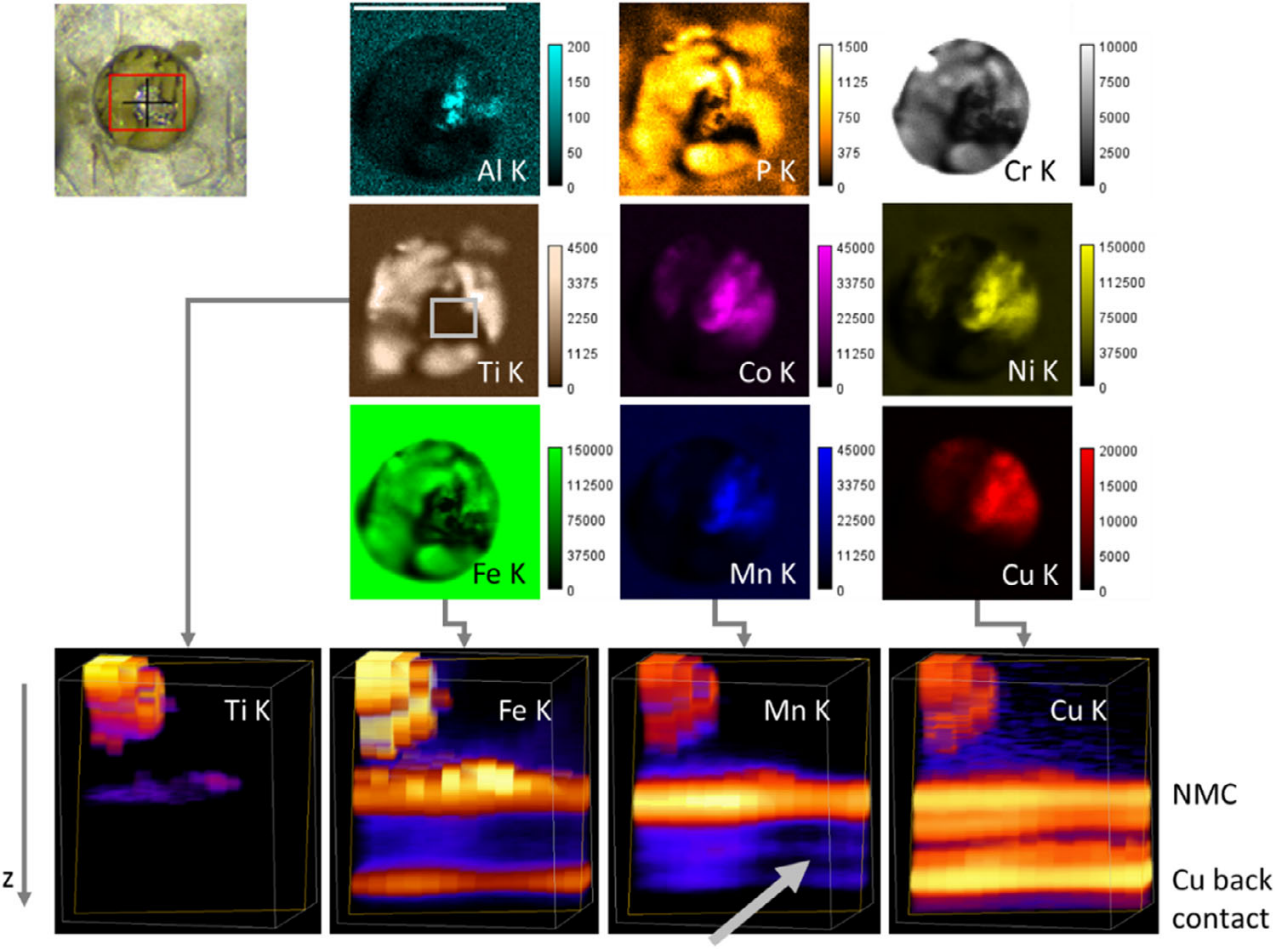
: top left: video image of the hole in the casing and window with crystallized electrolyte. Top right: Laboratory MXRF measurements of the cell post mortem (scale bar = 2 mm, net peak intensities in cps). The Fe K distribution highlights the cells casing, the Ti K, P K, Cr K and Fe K distributions show the location of the crystallized electrolyte, the Ni K, Co K, Mn K and Cu K distribution seem similar in the MXRF images; bottom: 3D rendering of the area marked in grey in the Ti K MXRF map measured with CMXRF. The grey arrow points to the gap forming between separator and carbon anode.

Post mortem but before disassembly, the cell window was imaged with micro‐XRF (MXRF), see **Figure** **5**. Without the polycapillary optic in the detection path, the K fluorescence from lighter elements such as Al and P can be imaged. The electrolyte seems to have penetrated to the outside of the cell and crystallized. Enhanced Cr, Fe and Ti intensities are visible in this material. As the excitation in the MXRF images is from the right and the detection from the bottom, information on deeper layers such as the NMC cathode is seen in the top right quadrant of the window.

In this static state, a full 3D volume CMXRF imaging measurement is feasible. A (650×450×720) µm^3^ volume was scanned with 50 µm lateral step size and 5 µm depth step size and the resulting XRF spectra deconvolved. In the four panels of Figure 5 bottom, the 3D distributions of the Ti, Fe, Mn and Cu K fluorescence are displayed. The 3D images show the gap between the separator and the carbon anode (grey arrow) as well as the lateral inhomogeneity with Ti (and the intense Fe areas) representing the crystallized electrolyte residues.

**Figure 6:**
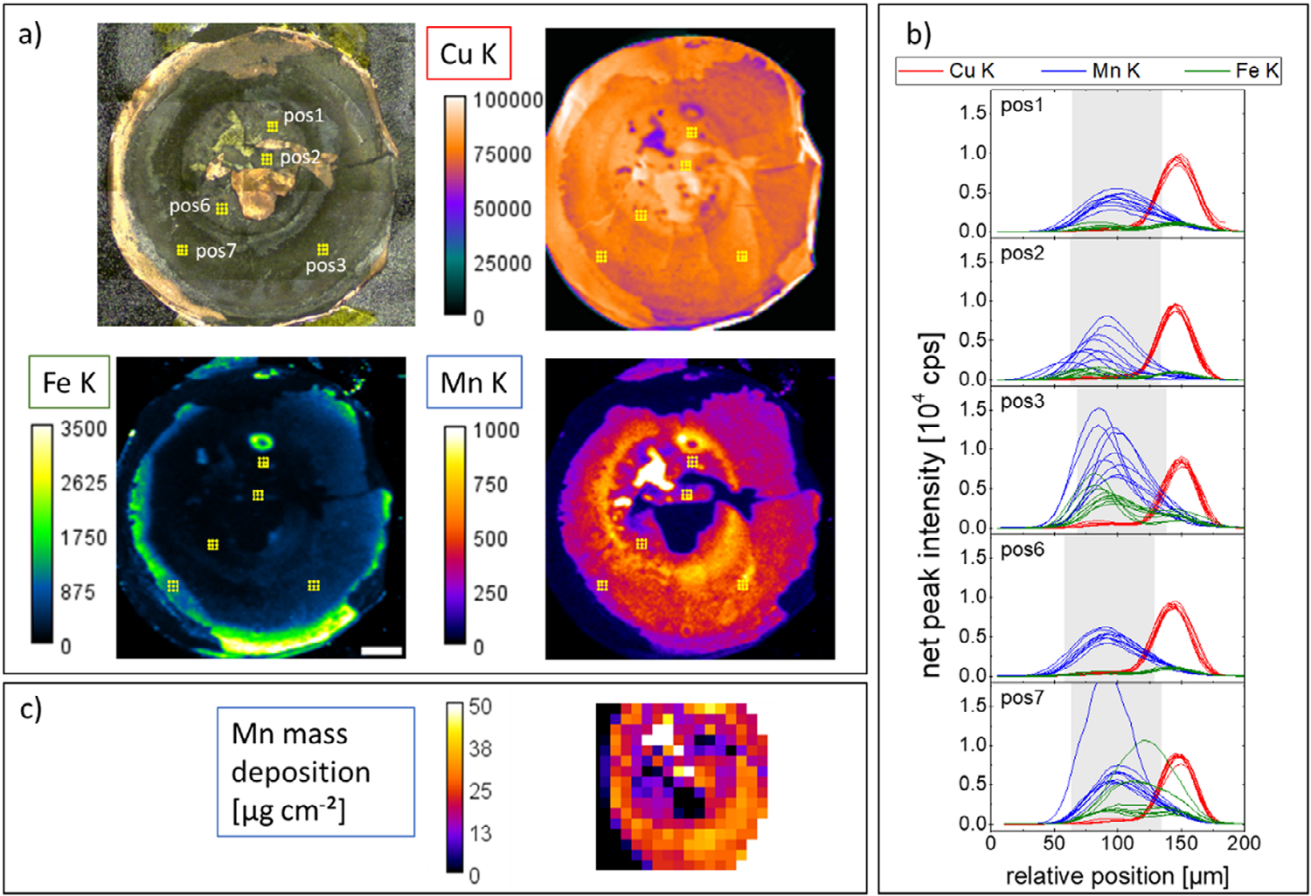
a) Video image and laboratory MXRF mapping of the carbon anode on top of the Cu foil (scale bar = 2 mm, net peak intensities in cps). b) In 5 areas (marked with yellow dots in a) 3×3 CMXRF depth profiles were measured. The Mn and Fe K signals vary significantly. c) Quantitative XRF measurements performed at the PTB laboratories of BESSY II result in absolute Mn mass depositions in µg/cm^2^ in the middle part of the anode. (scale bar = 2 mm).

After disassembly, the carbon anode on top of the Cu back contact was imaged both with MXRF and CMXRF. The MXRF map shows a Mn (and Co and Ni) enhancement in the carbon anode, see **Figure** **6**a). The intensity of the signal is highly heterogeneous laterally. The hole in the spacer is visible, with the highest deposition on Mn at the edge of this hole. In general, the area where the steel spacer put pressure on the stack of layers has a higher Mn signal. The reduced stack pressure in the area beneath the hole and/or the formed gap between the separator and the carbon anode might be the reason for the decreased Mn deposition in this area.

CMXRF measurements were conducted in five areas, in each area 3×3 depth profiles with 200 µm separation. The Mn K and Fe K fluorescence depth profiles (Figure 6b) show great variability with maximum Mn intensity values between 200 cps and 2000 cps, the highest values in areas outside the steel spacer hole. The shape of the depth profiles also shows delamination of the carbon anode layer from the Cu foil and variable shapes. Although the depth resolution of the used setup does not allow for the differentiation of the exact concentration profiles within the carbon anode, the different shapes hint to a heterogeneity in 3D.

Quantitative MXRF measurements were carried out at the four crystal monochromator (FCM) beamline of the PTB located at BESSY II. The results are consistent with the laboratory MXRF measurements. The black and white parts of the map are due to shadowing (left) and direct excitation (right) of the sample holder made of stainless steel. Using a monochromatic excitation of 8.8 keV and calibrated instrumentation, the Mn content can be quantified and related to the MXRF measurement. The uncertainty of the mass deposition is estimated to be 10%. Mn mass depositions of up to (36 ± 4) µg cm^−^^2^ can be observed at positions where the anode was intact. When excluding extremely high values (> 50 µg cm^−^^2^) and very low values (< 5 µm cm^−^^2^) to account for damaged areas of the anode, the data yields an average of 23 µg cm^−^^2^ with a standard deviation of 8 µg cm^−^^2^. With a cathode active mass loading of 13.5 mg cm^−^^2^ and given composition, this means that 1.3% of the Mn has migrated from the cathode to the anode during the life of the battery.

### 2.1 Toward CMXANES

**Figure 7:**
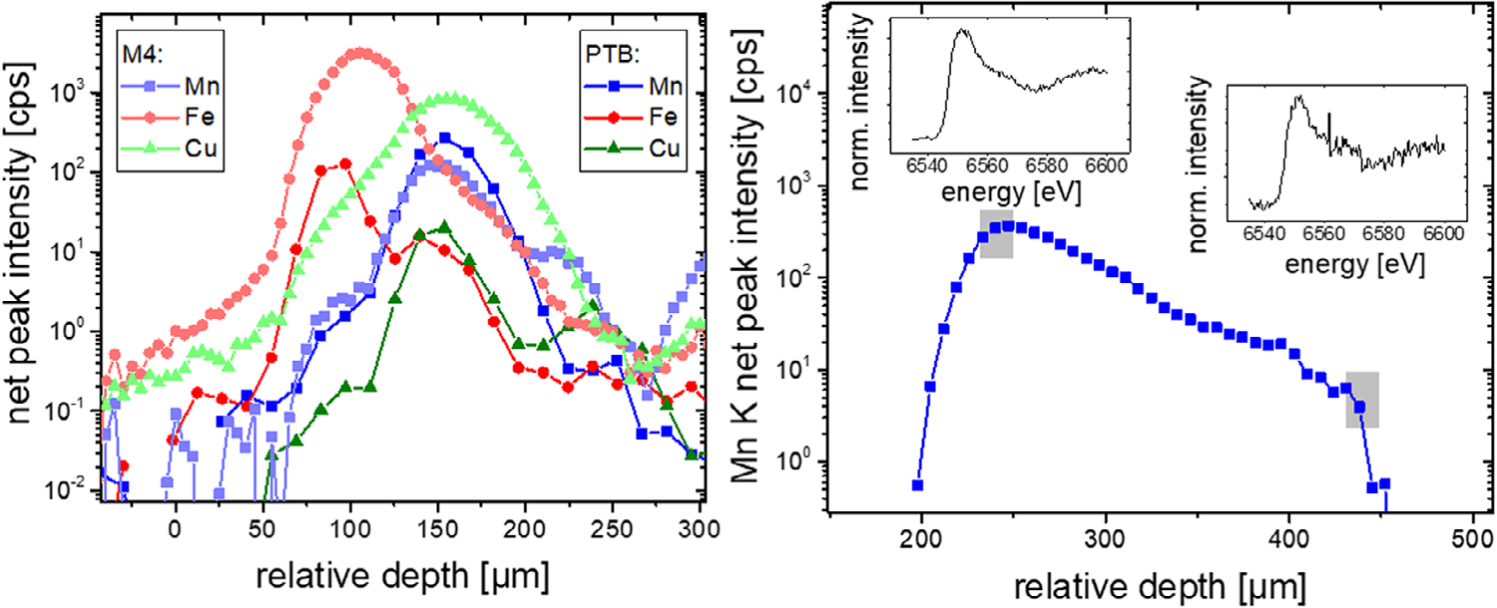
: left: Comparison of two depth profiling measurements on roughly the same lateral position with the laboratory M4 spectrometer and using synchrotron radiation at the PTB laboratory at BESSY II. The exemplary Mn K, Fe K and Cu K measurements demonstrate that the depth resolution is enhanced with monochromatic excitation and adapted optics, right: Example of CMXANES spectra obtained with 200 µm depth separation. The two Mn K CMXANES measurements (insets) were obtained at the two depth positions marked with grey in the Mn K fluorescence depth profile.

Additional to CMXRF, X‐ray absorption spectroscopy can be conducted in a confocal setup. For this purpose, a tunable X‐ray source is needed, thus, experiments must be performed at a synchrotron radiation facility. For a first proof‐of‐principle experiment of CMXRF on the intact cell, a setup was installed at the MiFo beamline. Table 1 lists a comparison of the FWHM and the maximum intensity value of depth profiles on thin foils for the two laboratory setups and the proof‐of‐principle experiment at PTB's MiFo beamline. The results show that with a monochromatic beam and an adapted polycapillary lens in front of the fluorescence detector the depth resolution capabilities can be improved by a factor of 2 compared to the used laboratory setups. This is visible also in the comparison of two depth profiles which were collected roughly at the same lateral position post mortem, see **Figure** **7** left.

The feasibility of CMXANES is presented in the right panel of Figure 7, on a different lateral position. Here, two Mn K XANES spectra were measured at positions with 200 µm separation. While the spectrum obtained deeper inside the sample suffers from high uncertainties, the general shape of the XANES spectrum is obtained. Measurements were performed in a low alpha week with ¼ of the typical ring current of BESSY II. Thus, experiments with the typical 300 mA ring current will facilitate depth resolved chemical speciation in the future.

## 3 Conclusions

We present elemental imaging of a NMC coin cell which is equipped with a graphite window and a steel spacer for pressure regulation. Using 60 CMXRF depth profiling measurements on the cycling cell during the course of 6 weeks and several thousands of cycles, changes in the elemental distribution are shown. Below the NMC cathode, TM diffusion is visible with a 4‐fold increase of Mn K fluorescence at the carbon anode within the first 3 weeks and approx. 200 cycles. We demonstrate that minute amounts of Mn (23 µg cm^−^^2^) can be located underneath a 6:2:2 NMC cathode (1600 µg cm^−^^2^ Mn mass deposition) and dynamic changes in the location of diffusing elements can be revealed. No further changes in the Mn deposition at the SEI can be discerned after 200 cycles. But a delamination of layers is evident. Post mortem and ex‐situ elemental analysis show a lower TM deposition in the area beneath the hole in the spacer. Here, we hypothesize that the delamination of layers which is possible because of the Ar bubble in the hole of the spacer is the main cause of the inhibition of further TM deposition. Connectivity between the layers is no longer given and reactions and degradation are slowed down or suspended. The higher Mn content observed in post mortem studies (see Figure 6) in the outer area of the electrode (where no delamination occurs) indicates that the degradation stopped only in the area of the CMXRF measurement. We do not assume that the mechanism has come to an end intrinsically.^[^ ^30^ ^]^ Future design of cells should minimize the spacer opening or completely change the coin cell geometry to achieve even more realistic battery systems.

Nevertheless, laboratory CMXRF spectrometers are well suited to non‐destructively investigate NMC coin cells with the only prerequisite being the necessity for an entrance window. On the one hand, long‐time depth profiling measurements are facilitated in operando. During cyclization, the layers of the cell move relative to each other, rendering full 3D investigations difficult due to long measurement times. Without cyclization, static measurements can be conducted enabling elemental imaging in 3D. As CMXRF spectrometers with X‐ray tubes as sources can be easily installed in production facilities, they enable, e.g., process control and defect monitoring on site. Contrary to synchrotron radiation investigations, laboratory setups render investigations over a long period of time feasible. The non‐destructive character of the measurements enables to monitor changes in the same cell at different points in the life time of the battery. This operando depth profiling option is fundamentally different from, e.g., SIMS measurements,^[^ ^31^, ^32^, ^33^ ^]^ where different cells are cycled for specific time periods and then investigated ex situ.

The addition of selective measurements at synchrotron radiation sources with calibrated instrumentation allows on the one hand reference‐free quantification of TM deposition on selected components after disassembly. The knowledge about the densities and compositions of the components of the stack is a necessary prerequisite for the quantification of CMXRF depth profiles^[^ ^17^ ^]^ which is planned for the future. On the other hand variable excitation energies facilitate the additional investigation of the chemical species of, e.g., TM depositions. As proof of principle we demonstrate that CMXANES can be conducted at specific locations in 3D. Experiments can be envisioned which combine long‐term laboratory CMXRF investigations with CMXANES measurements at a few time points at a synchrotron beamline setup without the necessity to stop the cyclization as a transfer between setups is straight forward. With adapted optics, the depth resolution can be decreased to values (10 µm to 20 µm, see Table 1) that facilitate the imaging of detailed elemental distributions within the individual layers such as the carbon anode of the battery in operando.

## 4 Experimental Section

### Materials

The developed LIB coin cells resemble the typical architecture as close as possible, depicted in Figure 1. Electrodes were produced by the MEET Battery Research Centre in their in‐house battery production line. The electrode paste consisting of active material, conductive carbon and binder with a solid content of 77% in N‐methyl‐2‐pyrrolidone was applied to the current collector foils. The stack consists of Cu back contact (10 µm), carbon anode (with an active mass loading of 7.9 mg cm^−2^, ratio of 95:0.5:4.5 of graphite, conductive carbon and CMC:SBR binder, 30% porosity), Celgard 2500 separator, NMC cathode (with an active mass loading of 13.5 mg cm^−2^, ratio of 95:2:3 of LiNi_0.6_Co_0.2_Mn_0.2_O_2_, conductive carbon and PVDF binder, 30% porosity) and Al current collector (15 µm), see Figure 1. Standard electrolyte LP572 (EC:EMC 3:7 1 M LiPF_6_ + 2% VC) was used.

To allow photon‐in/photon‐out experiments, a hole was introduced in the coin cell housing and sealed with 4 µm HOPG (highly oriented pyrolytic graphite) membrane, purchased from Optigraph GmbH. The selection of HOPG as entrance window was based on high transmission, mechanical stability, vacuum compatibility and general non‐toxicity. Additionally, a stainless steel spacer was inserted for better pressure distribution. The assembly was performed in Ar atmosphere. Cyclization parameters were listed in Table 2. The voltage minimum and maximum were changed three times to induce enhanced stress on the cell and reinforce degradation and different constant voltage holds were introduced. Post mortem, the cell was de‐crimped in air. There was no further processing or rinsing of the individual components.

### Methods

For all shown measurements in the presented figures, the parameters were listed inTable 3. Deconvolution of laboratory XRF spectra was conducted with an in‐house software SpecFit,^[^ ^18^ ^]^ resulting in net peak intensity values given in counts per second (cps). The graphic representation was conducted with ImageJ version 1.53q and OriginPro 2015.

*CMXRF Spectrometers at BLiX: BLiX Spectrometer and M4 Tornado*: Two laboratory CMXRF spectrometers were available. The first instrument was a commercial MXRF system (M4 tornado, Bruker Nano GmbH) equipped with a second polycapillary in front of a second SDD.^[^ ^24^ ^]^ It uses a Rh X‐ray tube with 50 kV/1 mA for excitation and the measurements were conducted in air or in 20 mbar vacuum atmosphere, see **Table** **3**. Post mortem MXRF and CMXRF experiments were conducted on the cell both post mortem and ex situ after disassembly of the individual layers.

**Table 3:** Measurement parameters for the images in the respective figures

Figure	Spectrometer	X‐ray source	Step Size [µm]	Measurement Time per Spectrum [s]	Measurement Mode
2	BLiX spectrometer	Cu, air	5	30 (real)	CMXRF depth profile
3	BLiX spectrometer	Cu, air	5	30/60/30 (real)	CMXRF depth profile
4	BLiX spectrometer	Cu, air	5 ‐10	30‐30 (real)	CMXRF depth profile
5	M4 spectrometer	Rh, air	25×25	0.25 (live)	MXRF 2D map
5	M4 spectrometer	Rh, air	50×50×5	30 (live)	CMXRF 3D volume
6a	M4 spectrometer	Rh, 20 mbar	100×100	1.5 (live)	MXRF 2D map
6b	M4 spectrometer	Rh, 20 mbar	200×200×5	30 (live)	CMXRF depth profile
6c	FCM @ BESSY II	8.8 keV	500×500	60 (real)	MXRF 2D map
7	MiFo @ BESSY II	10 keV	5	30 (live)	CMXRF depth profile
7	MiFo @ BESSY II	Mn K edge	0.5 eV	20/120 (live)	CMXANES

**Figure 8:**
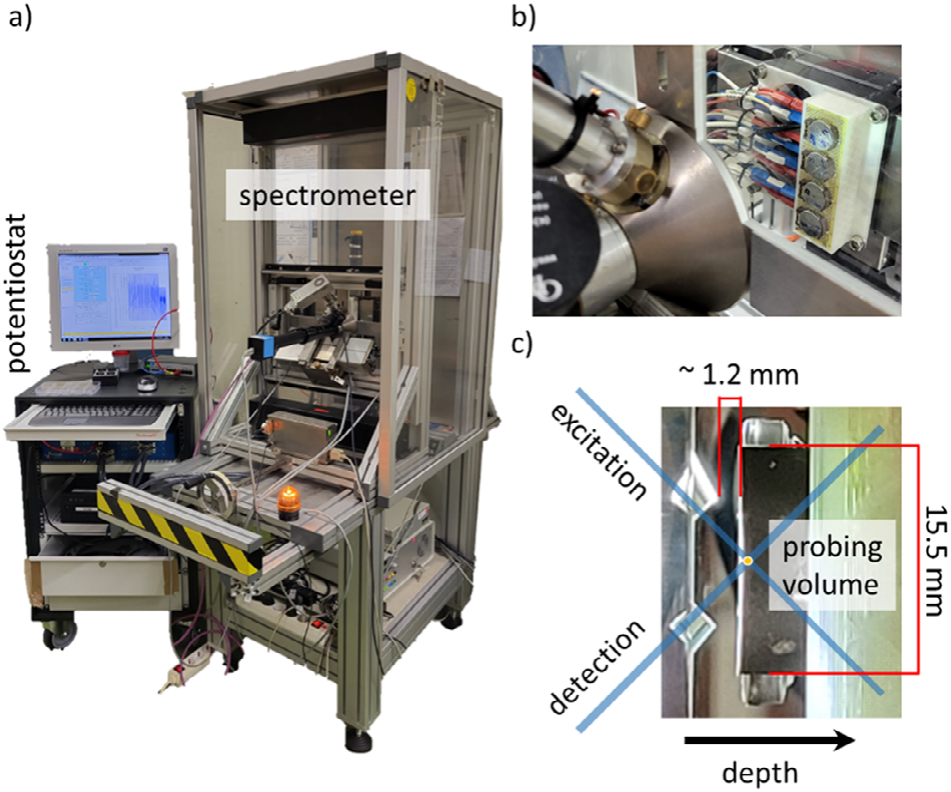
a) Photo of the BLiX CMXRF setup with potentiostat which was used for the operando measurements. b) Detail of the sample holder with 4 coin cells inside the measurement chamber. c) Picture from the side. The tips of both polycapillary lenses can be seen as well as the holder with the coin cell, demonstrating the small working distance.

The second instrument (BLiX spectrometer) was a CMXRF system,^[^ ^34^ ^]^ which allows for operando measurements, see **Figure** **8**. Here, a Cu microfocus X‐ray tube was used at 50 kV, 600 µA for an optimized excitation of the Mn K fluorescence. A cell holder was designed and 3D printed, which allows to cycle four cells simultaneously inside the spectrometer. In the holder, the cell surface was the highest position, allowing for the maximum depth‐range for depth profiling measurements.

*CMXRF Setup at MiFo Beamline @ PTB*: Additionally, a CMXRF setup was assembled at the MiFo beamline in the PTB laboratory at BESSY II. The beamline provides focused radiation in the tender X‐ray range and was still in the last phase of commissioning during the measurements.

The beamline allows to switch between a plane grating monochromator, optimized for photon energies from 1.5 keV to 3.5 keV, and a double crystal monochromator (DCM) with two Si(111) crystals providing X‐rays up to 10 keV. For the present measurements the beamline was operated using the DCM to provide monochromatized radiation at 10 keV for CMXRF and around 6.5 keV near the Mn‐K absorption edge for CMXANES measurements. A Kirkpatrick‐Baez (KB) optic focuses the incoming beam to a typical size of 20 µm x 20 µm. The coin cell was mounted on a 5‐axis manipulator in the XRF experimental end station under UHV conditions.^[^ ^35^ ^]^ For the confocal setup a polycapillary lens was mounted directly on the snout of the SDD housing. The custom‐designed, collimating polycapillary half lens with a 4.3 mm focal distance was obtained from Fischer GmbH.

Synchrotron CMXRF and CMXANES data has been deconvolved by means of a detector response function formalism using an in‐house Python routine. The resulting counts per incident energy were normalized to the incoming photon flux and exposure time.

*Quantitative MXRF Measurements @ PTB*: Reference‐free XRF quantification was performed on the four crystal monochromator (FCM) beamline for bending magnet radiation in the PTB laboratory at BESSY II.^[^ ^36^ ^]^ Reference‐free quantification relies on calibrated instrumentation and knowledge of all experimental and fundamental parameters (FP).^[^ ^37^ ^]^ The photon energy of the incident beam was set at 8.8 keV to excite all K‐shells of the elements of interest. The measured Mn Kα and Kβ fluorescence intensities were compared with a forward calculation using the Sherman equation, and the mass deposition of Mn adjusted using a fit routine. Due to the low mass deposition of high Z elements and high photon energies, the Mn mass deposition can be modelled as a thin homogeneous layer and independent of all other SEI components.

## Conflict of Interest

The authors declare no conflict of interest.

## Data Availability

The data that support the findings of this study are available from the corresponding author upon reasonable request.
